# Palliating Symptoms in Patients With Hepatocellular Carcinoma Involving the Inferior Vena Cava With External Beam Radiation Therapy

**DOI:** 10.7759/cureus.14107

**Published:** 2021-03-25

**Authors:** Peter Cooke, Kunal K Sindhu, Eric J Lehrer, Samuel Z Maron, Kenneth E Rosenzweig, Michael Buckstein

**Affiliations:** 1 Radiation Oncology, Icahn School of Medicine at Mount Sinai, New York City, USA

**Keywords:** hepatocellular carcinoma, inferior vena cava, external beam radiation therapy, right atrium

## Abstract

Introduction

Patients with hepatocellular carcinoma (HCC) involving the inferior vena cava (IVC) and/or right atrium (RA) often experience debilitating symptoms, including lower extremity edema, dyspnea on exertion, shortness of breath at rest, chest pain, and ascites, that impact quality of life. The efficacy of external beam radiation therapy (EBRT) in palliating these symptoms is unclear. Thus, we sought to assess the effectiveness of EBRT in the palliation of symptoms and treatment outcomes in this patient population.

Materials and methods

All patients with HCC that compressed or invaded the IVC, received EBRT, and had a two-month follow-up visit to assess clinical response at our institution between 2010 and 2018 were analyzed. Patient demographics and clinical features were retrieved from the electronic medical record. Local control, local progression-free survival, and overall survival (OS) were measured from the last day of EBRT and calculated using the Kaplan-Meier method.

Results

Twenty-six patients with invasion or compression of the IVC were identified, 11 of whom (42%) had involvement of the RA. The median follow-up was 3.6 months. Five patients (19%) were treated with stereotactic body radiation therapy (SBRT) (all with five fractions) and 21 patients (81%) were treated with fractionated radiation therapy (range 10-16 fractions), both to a median dose of 3,000 cGy (range 2500-4000 cGy for SBRT, 2500-3750 cGy for fractionated radiation therapy). Significant proportions of patients experienced symptomatic relief from peripheral edema (54%), dyspnea on exertion (57%), shortness of breath (83%), chest pain (67%), and ascites (25%) after receiving EBRT. Additionally, they experienced few toxicities, with zero experiencing grade three or higher toxicities. One-year and two-year local control rates were 11.5% and 7.7%, respectively, and the median local progression-free survival was 4.8 months. One-year and two-year OS rates were 38.4% and 38.4%, respectively.

Conclusions

Our results suggest that EBRT should be considered as a potential treatment option for patients with HCC invading or compressing the IVC with or without involvement of the RA. EBRT was very well-tolerated and effectively palliated a variety of symptoms in patients with advanced disease.

## Introduction

Hepatocellular carcinoma (HCC) is a leading cause of cancer mortality worldwide [1]. Approximately 3-4% of patients with HCC have invasion of the inferior vena cava (IVC) and/or right atrium (RA) at initial presentation or following treatment with transarterial chemoembolization (TACE) [2-4]. These patients have a poor prognosis and are at high risk of sudden death due to the development of a pulmonary embolism or acute heart failure [5]. External compression of the IVC, which can precipitate occlusion or thrombosis, can also have acutely life-threatening consequences [6]. IVC involvement may cause a number of symptoms, including lower extremity edema, dyspnea on exertion, shortness of breath at rest, chest pain, and ascites, that can significantly impair quality of life.

Systemic therapy is the standard of care for Barcelona Clinic Liver Cancer (BCLC) Stage C HCC [7]. For patients with HCC invading the IVC, however, an international consensus regarding the standard of care is lacking. Surgical resection has shown varied results in extending lifespan and quality of life, but is often avoided due to a high risk of complications [8-11]. The efficacy of TACE has also been retrospectively examined in small cohort studies that have shown mixed results, in terms of overall survival (OS) and local control (LC), as compared to surgical outcomes [2, 12-14]. Some guidelines, including those of the National Comprehensive Cancer Network, suggest external beam radiation therapy (EBRT) as a first-line treatment of advanced HCC. Others, including those issued by the European Association for the Study of the Liver, deprioritize EBRT as a treatment option for these patients [15]. Despite ongoing debate, the use of EBRT is rising in patients with HCC and invasion of the IVC due to the treatment’s ability to impact tumor burden on/in major vessels. For patients with HCC compressing the IVC, stenting is often the treatment option of choice [16].

The outcomes of patients with HCC invading the IVC who have received EBRT have been reported in several small patient cohorts in Asia. A meta-analysis of these studies revealed a one-year OS of 53.6%, comparable to the one-year OS of TACE alone [13-16]. While limited toxicity was reported during treatment, none of the studies in this meta-analysis evaluated EBRT’s efficacy in palliating symptoms secondary to advanced HCC. There is even more limited research on outcomes in patients with HCC and right atrial involvement who receive EBRT: one cohort study of 10 patients reported a one-year OS of five months, but the efficacy of the treatment in palliating symptoms was not reported [17]. Thus, in order to assess outcomes and the effectiveness of EBRT in palliating symptoms in this population, we examined patients with HCC and involvement (compression or invasion) of the IVC with and without involvement of the RA who received EBRT at our institution.

## Materials and methods

All patients with HCC that compressed or invaded the IVC with or without involvement of the RA and received EBRT at our institution between 2010 and 2018 were identified. Patients with a two-month post-EBRT follow-up visit to assess clinical response were included in this analysis. EBRT targeted the intrahepatic tumor burden and additional tumor thrombi. Patient demographics and clinical features were retrieved from the electronic medical record. Symptoms prior to EBRT, treatment toxicity (CTCAE v4.0), and response to treatment were noted. LC and OS were measured from the last day of EBRT and were calculated using the Kaplan-Meier method. This study was approved by our Institutional Review Board (IRB).

## Results

We identified 26 patients who met our inclusion criteria (Table 1). Twenty-four patients (92%) had invasion of the IVC and two patients had (8%) compression of the IVC. Eleven patients (42%) had involvement of the RA. The median follow-up for all patients was 3.6 months.

**Table 1:** Demographics and staging of the 26 patients included in this analysis EBRT = External beam radiation therapy, SD = standard deviation, HBV = hepatitis B virus, HCV = hepatitis C virus, BCLC = Barcelona Clinic Liver Cancer staging system, IVC = inferior vena cava, RA = right atrium, HCC = hepatocellular carcinoma, Y90 = Yttrium-90 radiation therapy, TACE = transarterial chemoembolization, RFA = radiofrequency ablation

Variable	N	Percent
Age, median (SD)	65 (15)	
Sex		
Male	24	92.3
Female	2	7.7
HCC etiology		
HBV	3	11.5
HCV	14	53.8
Alcoholism	2	7.7
Cryptogenic	7	26.9
Child Pugh Class		
5A	9	34.6
6A	9	34.6
7B	4	15.4
8B	3	11.5
9B	1	3.8
BCLC Class		
A	1	3.8
B	4	15.4
C	21	80.1
Intrahepatic tumor number		
Single	5	19.2
Multiple	21	80.1
Extension of thrombus		
IVC compression	2	7.7
IVC invasion	13	50.0
IVC + RA invasion	11	42.3
Lesion size involving IVC ± RA, median in centimeters (SD)	8.5 (4.9)	
Additional liver-directed therapy		
Y90 concurrent with EBRT	10	38.4
Y90 post-EBRT	4	15.4
TACE pre-EBRT	8	30.7
TACE post-EBRT	5	19.2
RFA pre-EBRT	4	15.4

**Figure 1:**
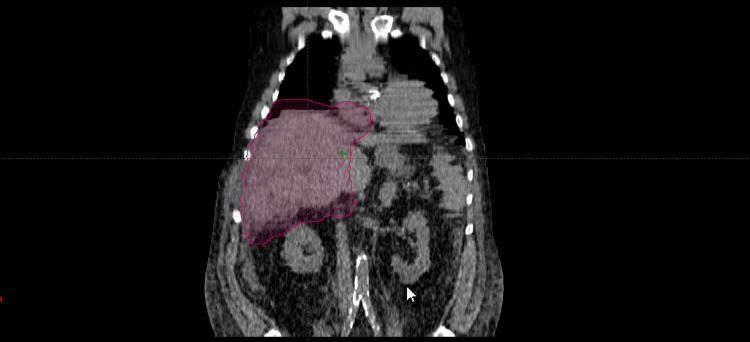
The planning target volume in a patient with hepatocellular carcinoma invading into the inferior vena cava and extending into the right atrium who received external beam radiation therapy

Five patients (19%) were treated with stereotactic body radiation therapy (all with five fractions) and 21 patients (81%) were treated with fractionated radiation therapy (range 10-16 fractions), both to a median dose of 3,000 cGy (range 2500-4000 cGy for stereotactic body radiation therapy (SBRT), 2500-3750 cGy for fractionated radiation therapy). The median planning target volume (PTV) was 454.1 cubic centimeters (cc) (range 89.1-413.2 cc for patients who received SBRT, 97.6-2736.7 cc for those who received fractionated radiation therapy). Nine patients (35%) had a PTV of greater than 500 cc, while four patients (15%) had a PTV of greater than 1500 cc (Figure 1).

Twenty-four patients received systemic therapy, 19 (73%) of whom received sorafenib and five (19%) of whom received nivolumab. Two patients did not receive systemic therapy. In addition, most patients received additional liver-directed therapy preceding or following EBRT. Ten (38%) patients received Y90 chemoembolization concurrent with EBRT and four patients (15%) received Y90 following EBRT. Eight patients (31%) received TACE prior to EBRT, while five patients (19%) received TACE therapy following EBRT. Four patients (15%) underwent radio-frequency ablation (RFA) prior to EBRT.

**Figure 2:**
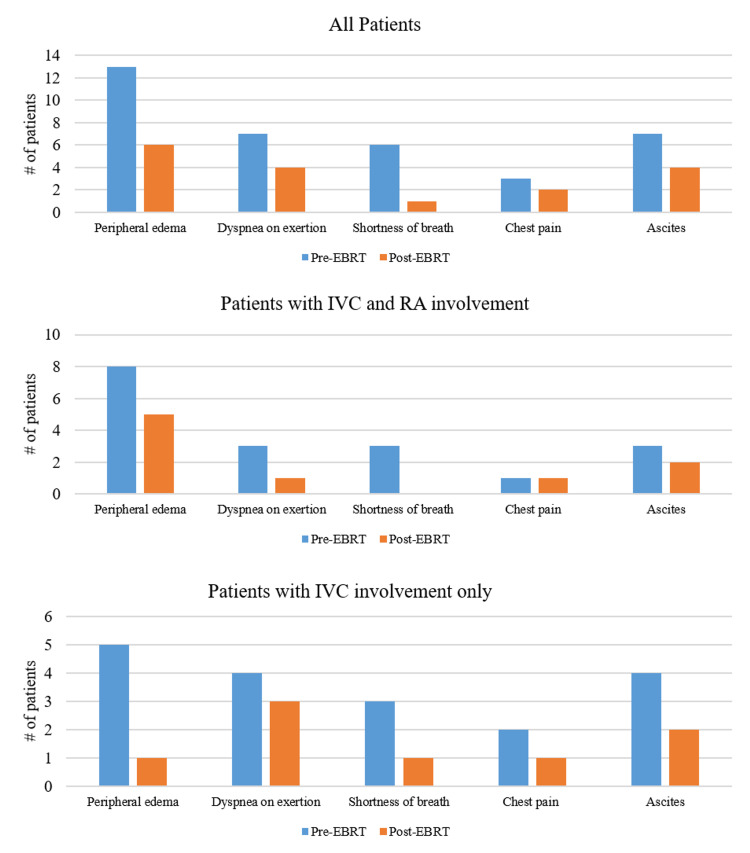
Patients with symptoms before and after external beam radiation therapy IVC = inferior vena cava, RA = right atrium, EBRT: External beam radiation therapy.

Five prominent symptoms were analyzed for palliation (Figure 2). Prior to EBRT, 13 patients (50%) presented with peripheral edema, seven (27%) with dyspnea on exertion, six (23%) with shortness of breath at rest, three (12%) with chest pain and four (15%) with ascites. Among all patients included in this analysis, seven of 13 patients (54%) with peripheral edema, four of seven patients (57%) with dyspnea on exertion, five of six patients (83%) with shortness of breath, two of three patients (67%) with chest pain, and one of four patients (25%) with ascites experienced an improvement in their symptoms after EBRT at a median follow-up of 2.2, 1.9, 1.9, 1.5, and 2.2 months, respectively. Responses were durable: the median interval of symptomatic relief after EBRT was 6.5, 6.2, 5.8, 4.3, and 3.8 months, respectively. Among patients with both IVC and RA involvement, two of three patients (67%) with dyspnea on exertion and three of three patients (100%) with shortness of breath reported symptomatic relief after EBRT. In contrast, among patients with IVC invasion without RA involvement, four of five patients (80%) with peripheral edema, two of three patients (67%) with dyspnea on exertion, and two of three patients (67%) with shortness of breath at rest reported a reduction in their symptoms after EBRT. Both patients with compression of the IVC experienced an improvement in peripheral edema after EBRT.

**Figure 3:**
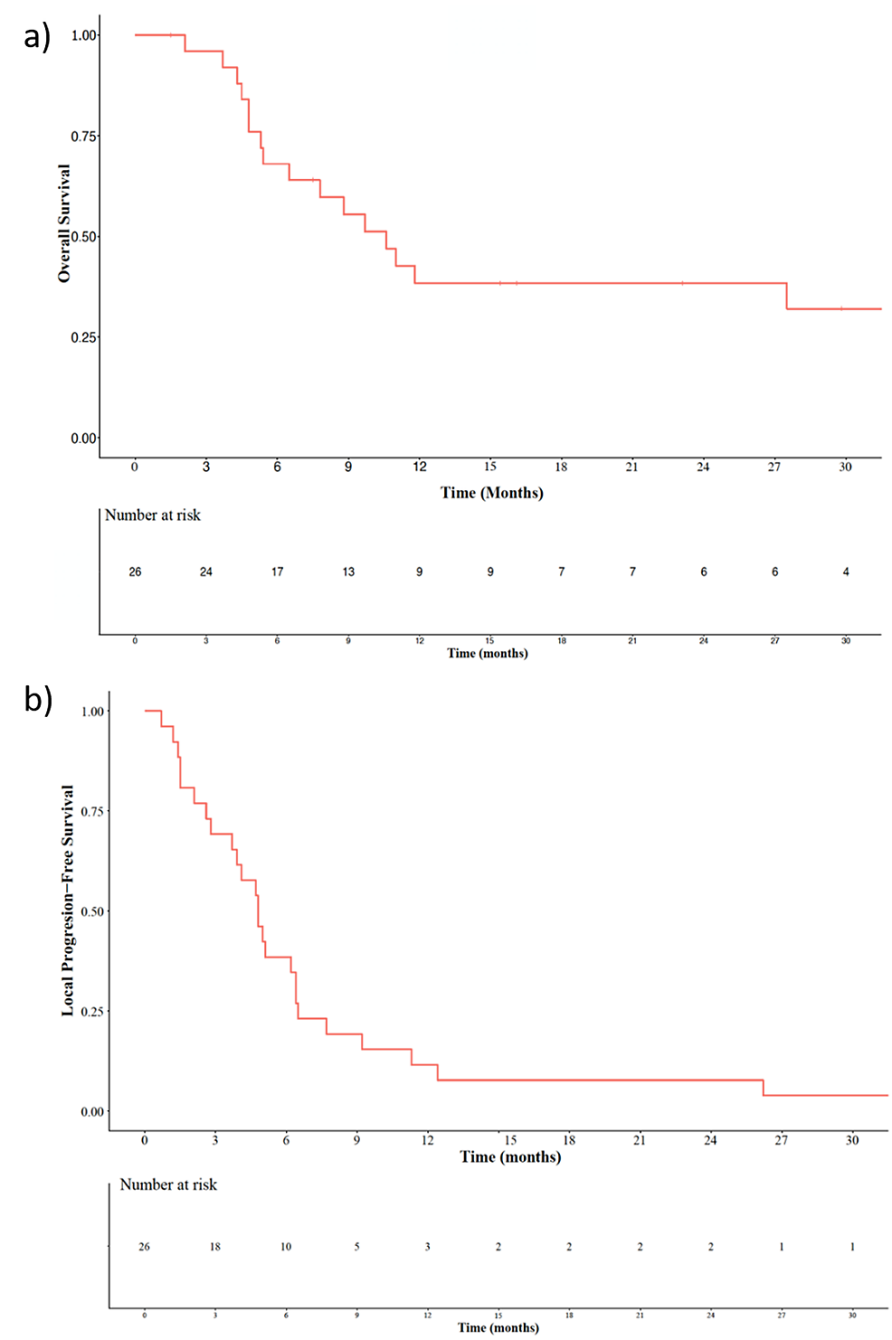
Kaplan-Meier curves for a) overall survival and b) local progression-free survival for the twenty-six patients cohort

One- and two-year local control rates for the 26-patient population were 11.5% and 7.7%, respectively. The median local progression-free survival was 4.8 months. One- and two-year OS rates were 38.4% and 38.4%, respectively. The median OS was 9.2 months (Figure 3).

Nine of the 26 (35%) examined patients did not experience any toxicity during EBRT. Fourteen patients (54%) had grade one toxicities: one had gastroesophageal reflux disease (GERD), four had fatigue, two had dyspepsia, two had diarrhea, two had abdominal pain, and two had anorexia. Three patients (12%) had grade two toxicities: two had GERD, two had fatigue, one had dyspepsia, one had diarrhea, and one had anorexia. Zero patients experienced grade three or higher toxicities.

## Discussion

Patients with advanced HCC invading or compressing the IVC, with or without involvement of the RA, are challenging to treat. They generally experience poor outcomes and may experience debilitating symptoms that can severely impact quality of life. At this stage, clinical treatment paradigms are still evolving. However, when weighing treatment options, symptom relief and quality of life should prominently figure into the decision. Due to the high-risk location of tumors invading the IVC and RA, the non-invasive nature of EBRT renders it a valuable palliative treatment option [18].

Systemic therapy is often used in advanced cases of HCC. The phase three SHARP (Sorafenib HCC Assessment Randomized Protocol) trial evaluating the efficacy of sorafenib in patients with Child Pugh class A HCC resulted in a median OS of 10.7 months versus 7.9 months compared to placebo [19]. In a sub-analysis of the SHARP trial, Bruix et al. found that patients with HCC and major vascular involvement treated with sorafenib had a median OS of 8.1 months [20]. More recently, a phase III trial of patients with unresectable HCC who had not received prior systemic therapy found that combination therapy with atezolizumab and bevacizumab offered superior outcomes than sorafenib [21].

TACE is another treatment option often pursued in this patient population, and reported outcomes have varied widely. In a 2013 study, Wang et al. reported a median OS of just 4.5 months for 45 HCC patients with IVC invasion who received therapy with TACE [14]. In contrast, in a 2019 study of 18 patients with HCC invading the RA who received TACE, Zhu et al. reported a one-year OS of 50% and a three-year OS of 16.7% [13].

Surgical resection of the tumor thrombus in patients with HCC invading the IVC ± RA has also been studied in small cohorts treated. Kokudo et al. examined 71 patients who received surgical resection of HCC invading the IVC, and reported a one-year OS of 63.2% and a median OS of 16.4 months [22]. Wakayama et al. reported a recurrence-free survival of 3.8 months and median OS of 15.3 months in a cohort study of 13 patients [8]. In another cohort study of 25 patients, Wang et al. reported a one-year OS of 68% and median OS of 19.0 months [14]. However, in each study, only patients with Child-Pugh Class A cirrhosis were eligible for surgery. This necessary constraint, intended to limit surgical morbidity and mortality, prevents many patients with HCC and major vascular involvement from pursuing surgical intervention in practice. Additionally, the technical difficulty of surgery limits this option to patients treated at specialized surgical centers. Surgery was not pursued in this cohort of patients for a number of reasons, including patient comorbidities, patient preferences, and the technical difficulty of the proposed surgery.

EBRT, by contrast, is non-invasive and available as a treatment option to the vast majority of these patients. In the cohort of patients examined in this study, EBRT resulted in a marked palliation of symptoms (25-83%). Palliation of symptoms by EBRT was durable, as well. Furthermore, it was very well tolerated, with no patients experiencing worse than grade two toxicities and nine of 26 patients experiencing no toxicities at all. These results suggest that EBRT is a valuable palliative treatment option for this patient population.

There were several limitations to this study. Both the limited sample size and retrospective nature limits its generalizability and the ability to draw definitive clinical conclusions. The inclusion criteria requiring at least two months of clinical follow-up, which was required to assess for symptomatic relief, may have excluded patients who followed up at other institutions or passed away shortly after EBRT. Furthermore, patients in our cohort received different systemic therapies, which may have confounded our results. Larger cohorts of patients must be analyzed to properly discern the impacts of various systemic therapies. Additionally, only five patients received SBRT in this study, a cohort too small from which to draw conclusions about its efficacy. Further research is needed to discern the impact of SBRT versus fractionated radiation therapy in this patient population.

## Conclusions

Our results suggest that EBRT should be considered as a palliative treatment option for patients with HCC invading or compressing the IVC with or without involvement of the RA. EBRT was very well-tolerated and effectively palliated a variety of symptoms in the patients with advanced hepatocellular carcinoma included in this study. However, this study is limited by its small sample size and retrospective nature.
